# Plasmonic nanoparticles tuned thermal sensitive photonic polymer for biomimetic chameleon

**DOI:** 10.1038/srep31328

**Published:** 2016-08-09

**Authors:** Yang Yan, Lin Liu, Zihe Cai, Jiwen Xu, Zhou Xu, Di Zhang, Xiaobin Hu

**Affiliations:** 1 State Key Laboratory of Metal Matrix Composites, Shanghai JiaoTong University, Shanghai 200240, People’s Republic of China; 2 Guangxi Key Laboratory of Information Materials, Guilin University of Electronic Technology, Guilin 541004, People’s Republic of China

## Abstract

Among many thermo-photochromic materials, the color-changing behavior caused by temperature and light is usually lack of a full color response. And the study on visible light-stimuli chromic response is rarely reported. Here, we proposed a strategy to design a thermo-photochromic chameleon biomimetic material consisting of photonic poly(N-isopropylacrylamide-co-methacrylic acid) copolymer and plasmonic nanoparticles which has a vivid color change triggered by temperature and light like chameleons. We make use of the plasmonic nanoparticles like gold nanoparticles and silver nanoparticles to increase the sensitivity of the responsive behavior and control the lower critical solution temperature of the thermosensitive films by tuning the polymer chain conformation transition. Finally, it is possible that this film would have colorimetric responses to the entire VIS spectrum by the addition of different plasmonic nanoparticles to tune the plasmonic excitation wavelength. As a result, this method provides a potential use in new biosensors, military and many other aspects.

The color of most animals is relatively fixed, but some animals are capable of undergoing rapid, physiological color change which allows them to display different colors and patterns in response to changing environmental contexts^1^. Chameleons are well known for their striking capacity to rapidly shift the skin colors against surroundings such as temperature and surrounding colors^2^,^3^,^4^,^5^,^6^. It is the way for chameleon to disguise and protect themselves, adapt to circumstances and communicate with each other. Inspired by the chameleons, the biomimetic chameleon materials which have thermo- and photo-stimuli chromic responsive behaviors to surroundings will be of great benefits on biosensors, military camouflage and stealth, information transmission and so on^7^,^8^,^9^. The essential factor of biomimetic chameleon is to realize the automatic color change effect on stimuli triggered by surroundings temperature and reflective light (color), which can be enabled by the thermal and photochromic materials like RhodamineB^10^,^11^,^12^, poly(alkyloxide) copolymers^13^,^14^, silver halides^15^ and azobenzene polymers^16^. However, these known materials haven’t either a full-color responding or a visible light-stimuli chromic behavior. Marel and Milinkovitch indicated that the color change effect of chameleons was attribute to the active tuning of an ordered guanine nanocrystals lattice within their dermal iridophores^17^. This color change mechanism is the same as artificial photonic crystal (PC) which shows various colors by changing the photonic band gap (PBG)^18^. Many reports have proved the vivid colorimetric responses of the artificial PC to pH^19^, ions^20^, CO_2_^21^ and protein^22^ etc. What’s more, Zhang^23^ and Takeoka^24^ used the poly(n-isopropylacrlamide) (PNIPAm) microgels with photonic structure to realize the thermochromic response. These reports have indicated that the thermal response of PNIPAm is ascribed to the molecular conformation transformation from intermolecular to intramolecular interaction (hydrogen bonding) with the temperature going over the lower critical solution temperature (LCST), causing the reversible volume shrinkage simultaneously^24^,^25^,^26^,^27^. Thus, the PBG shift in concomitancy with volume change has been used to exhibit rapid, colorful response to temperature. However, these “soft photonic crystals” are not sensitive enough for temperature, not to mention the infrared and the visible light.

On the other hand, there are lots of reports to take use of the localized surface plasmon resonance (LSPR) of plasmonic nanoparticles for enhancement of the light-to-thermal conversion^28^,^29^,^30^,^31^,^32^,^33^. Au and Ag nanoparticles (AuNPs, AgNPs) are usually added into the thermosensitive PNIPAm film to increase the light absorption and the light-to-thermal conversion efficiency of the visible light with obviously temperature variation in film. Therefore, it’s believable that the plasmonic nanoparticles can be used to improve the sensitivity of the PNIPAm film to diversified stimuli, especially the visible light.

In this article, we present polymeric films coupling inverse opal structure with plasmonic nanoparticles such as AuNPs^34^ and AgNPs^35^ for biomimetic chameleon shown in Fig. 1, which is sensitive to the surrounding temperature, the infrared light (NIR) and the visible light (VIS). Firstly, the thermosensitive film has a full-color response to the variation of temperature due to the PBG shift resulting from the reversible volume shrinkage. Secondly, the NIR and VIS irradiation can also give rise to the improved allochroic response by the way of coupling the thermosensitive inverse opal film with plasmonic nanoparticles. The photo-stimuli chromic mechanism of the film is due to the light-to-thermal conversion effect, which leads to the film temperature increase and the film volume decrease. Moreover, the further results demonstrate that the themal- and photo-stimuli chromic response is attributed to the molecular conformation transformation of PNIPAm when the film temperature increases to induce conformation rotation from intermolecular hydrogen bond (state 1) into intramolecular hydrogen bond (state 2). As a concomitant result, the reasonable inference is that the thermosensitive film can exhibit different response to temperature, NIR and VIS below and above LCST because of the variation of polymeric physical properties such as density, volume and refractive index.

## Results and Discussion

### Thermal-stimuli chromic response

**Figure 2:**
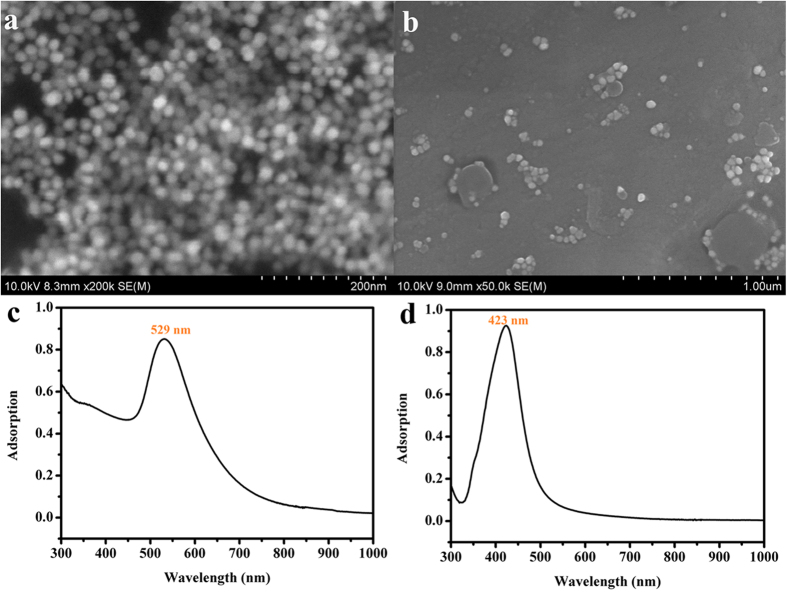
SEM images of the (**a**) AuNPs and (**b**) AgNPs and the absorption spectra of (**c**) AuNPs (529 nm) and (**d**) AgNPs (423 nm).

To develop sensitively thermoresponsive polymer, we prepared a copolymer hydrogel film of n-isopropylacrlamide (NIPAm) and methacrylic acid (MAAc) with and without AuNPs and AgNPs. The obtained P(NIPAm-co-MAAc) copolymer is shortened to be PNcM in the next depiction. The preparation of copolymer and the synthesis of AuNPs and AgNPs are shown in the experimental section. And the SEM images of the well-distributed AuNPs and AgNPs are described in Fig. 2a,b. The absorption peak of AuNPs is 529 nm and the one of AgNPs is about 423 nm shown in Fig. 2c,d. The peak position of the PBG for the films is obtained by Bragg’s equation^36^


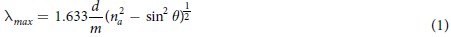


where d is the diameter of the colloidal particles used which is proportional to the length of the films, m is the order of the Bragg reflection, n_a_ is the refractive index of the photonic crystal, and θ is the angle measured from the normal to the plane of the photonic crystal. Under normal incidence, equation (1) can be rewritten as follows:


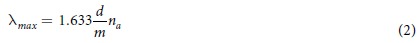


**Figure 3:**
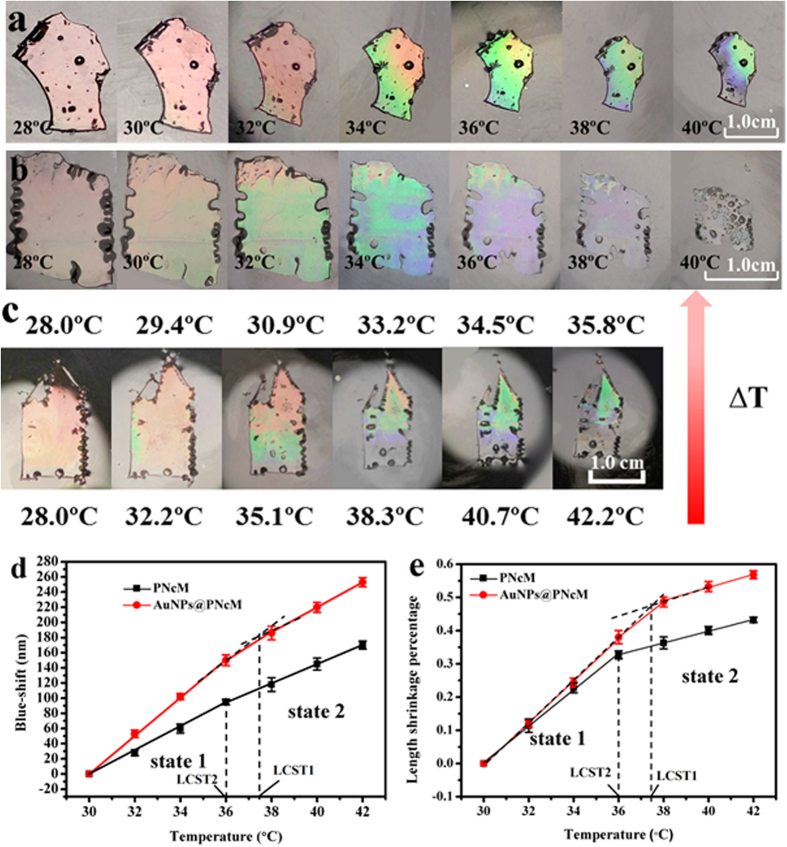
Thermo-stimuli chromic responses of both (**a**) PNcM and (**b**) AuNPs@PNcM films of color change, (**c**) PNcM film dependent on temperature gradient of color change, (**d**) blue shift and (**e**) length shrinkage percentage dependent on temperature. The temperature gradient experiment was conducted by injecting 50 °C DI water to the bottom of the PNcM film (the volume of injection water varied from 0, 0.6, 1.2, 1.8. 2.4, 3.0 ml); the initial temperature at the bottom was tested by infrared thermometer and the temperature at the top was calculated.

The inverse opal PNcM film displays the thermochromic response to temperature variation shown in Fig. 3a,b. And the colorimetric response of PNcM film to temperature gradient is shown in Fig. 3c. This kind of thermochromic response is sensitive and quick, so Fig. 3a,b are obtained in temperature equilibrium water and Fig. 3c is obtained instantaneously in temperature nonequilibrium water. For both of films with and without AuNPs soaking in water with different temperature, the vivid color change demonstrates that both films have a blue shift of PBG accompanied by volumes shrinkage obviously. In order to depict the sensitive thermochromic response clearly, the responsive sensitivity of the film is defined to be the blue shift of PBG dependent on temperature. Figure 3d shows clearly that the responsive sensitivity of both films increases with increasing temperature, but the film with AuNPs is more sensitive. It is notable that both films display a transition in blue shift dependent on temperature. Since the film thickness is far less than the film length, the film volume contraction can be represented by the film length shrinkage. In corresponding with Fig. 3d, the length shrinkage percentage of films dependent on temperature in Fig. 3e can also demonstrates the thermoresponsive sensitivity according to the equation 2 and the physical properties variation of films below and above LCST. Figure 3e shows more clearly that there are two states for both films due to the conformation transition, which results in different physical properties. Comparing two states of both films, the state 2 has smaller slope, which means the smaller variation of the length shrinkage percentage dependent on temperature above LCST. The reason can be inferred that both films in state 2 have bigger densities and rigidities due to intramolecular interaction inducing more difficult conformation rotation and volume shrinkage, thus the thermoresponsive behaviors of both films are relatively less sensitive but stronger reflective (see the Supplementary Fig. S1). Figure 3e also shows apparently that the film with AuNPs is more thermosensitive than the pure PNcM film because of its bigger slope, the reason of which is that these AuNPs possess nearly 20 times higher density and 10 times lower specific heat capacity than the polymer. The density is 1.0 g/cm^3^ of the PNcM as calculated and 19.32 g/cm^3^ of the AuNPs. The specific heat capacity is about 1.4~1.5 J/g·K^37^ of the PNcM and 0.128 J/g·K of AuNPs. When reaching to the same temperature, the AuNPs@PNcM film can absorb more energy from surroundings than the PNcM film. The excess energy is provided to the conformation transition which leads to the larger shrinkage in volume of the AuNPs@PNcM film. More importantly, Fig. 3e shows clearly that AuNPs can tune the LCST up from about 36 °C of the film without AuNPs to about 37.4 °C of the film with AuNPs. The reason may be presumed that AuNPs like “anchors” can restrict polymer chains around them rotating, thus inducing the bigger demand of thermal energy for the film with AuNPs to tune the polymer conformation and the state of LCST.

### NIR-stimuli chromic response

**Figure 4:**
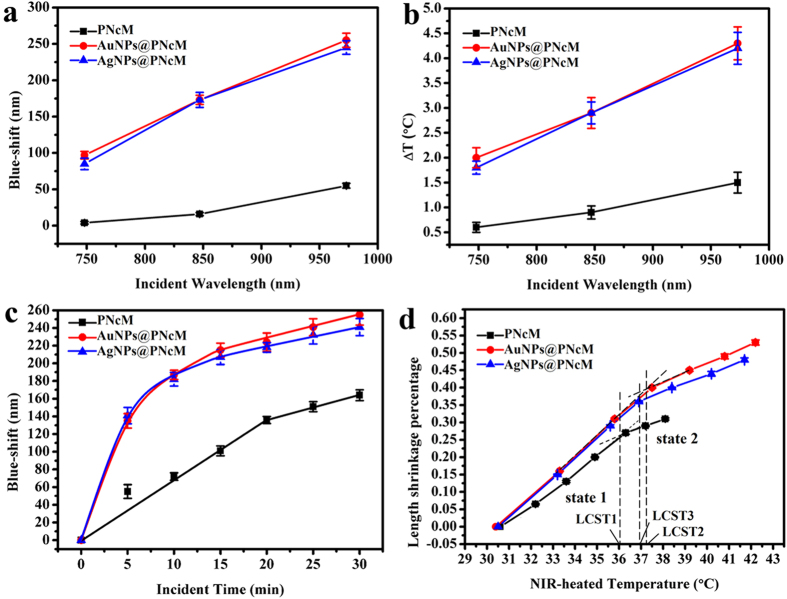
NIR-stimuli chromic responses of PNcM, AuNPs@PNcM and AgNPs@PNcM films of (**a**) blue shift and (**b**) temperature increment under individually 748, 847 and 973 nm NIR illumination for 10 min, then (**c**) blue shift and (**d**) length shrinkage percentage under 973 nm NIR illumination for 0, 5,10, 15, 20, 25, 30 min, respectively.

In view of the strong light-to-thermal conversion effect of the NIR, three kinds of wet films are used to examine their photoresponsive behaviors under irradiation of 748, 847 or 973 nm monochromatic light with the same irradiance, respectively. Three films are the PNcM films with nothing, AuNPs and AgNPs addition. In order to exclude interferences from water evaporation and heat transfer, all films are soaked in less water to form a thin water film above and the NIR lamp is focused on the objective films. Figure 4a shows the photoresponsive sensitivity of three films through the blue shift of PBG dependent on incident wavelength at the same irradiation time of 10 minutes. The obvious result is that the longer incident wavelength leads to the bigger blue shift of PBG. Meanwhile, plasmonic nanoparticles make films have stronger blue shifts than that of the film with nothing addition. The believable results in Fig. 4b are that the longer incident NIR has the stronger ligth-to-thermal conversion effect to cause a higher film temperature increase. Besides, the films with AuNPs and AgNPs have much higher temperature than that of the film with nothing addition under the same wavelength NIR irradiation for the same time. The Supplementary Fig. S2 describes the adsorption spectrums of PNcM, AuNPs@PNcM and AgNPs@PNcM films without photonic crystal structure. It is clearly that the nanoparticles have no obvious enhancement of the NIR adsorption. The reason why films with nanoparticles can get a higher photosensitivity is that the AuNPs and AgNPs with lower specific heat capacity and higher thermal conductivity could obtain higher temperature than the polymer at the same irradiating time and irradiance. And the nanoparticles can act as heaters to heat up the polymer around. Figure 4c is much better to demonstrate the dynamical impact of 973 nm NIR on the photo-stimuli sensitivity of the three films (color images in Supplementary Fig. S3) by means of the blue shift of PBG dependent on time. It is fully observed that the longer irradiation time caused the bigger blue shift and higher temperature shown in Supplementary Fig. S3. Like the responding to temperature, Fig. 4d shows again that the higher film temperature from the longer irradiation time is one-to-one corresponding to the larger film length shrinkage percentage. And three films without exception present two states with increasing temperature, especially for both plasmonic films with more obvious photo-responses and higher LCST points. The reason is also the same anchoring effect of plasnomic nanoparticles to surrounding polymer chains. And when the films are heated by NIR to the same temperature increasement, just like the themal-stimuli chromic response, the AuNPs@PNcM film can obtain more energy from the surroundings than the PNcM film to achieve the larger length shrinkage.

### VIS-stimuli chromic response

**Figure 5:**
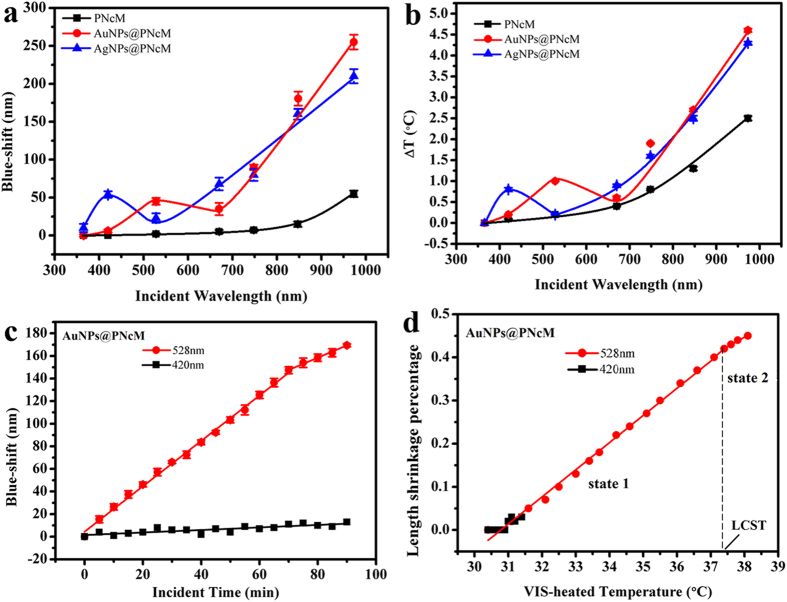
VIS-stimuli chromic responses of PNcM, AuNPs@PNcM and AgNPs@PNcM films of (**a**) blue shift and (**b**) temperature increment under individually 365, 420, 528, 670, 748, 847 and 973 nm light illumination for 10 min, then only the AuNPs@PNcM film of (**c**) blue shift and (**d**) length shrinkage percentage under 528 and 420 nm illumination for time from 0 to 90 min with 5 min interval.

Since the VIS is the most important and usual stimuli impacting on the biomimetic chameleon, the VIS-stimuli chromic behaviors and the plasmonic tuning effects are detailedly examined by the same way of the NIR experiments for all three kinds of films with nothing, AuNPs and AgNPs addition. The Supplementary Fig. S4 and Fig. 5a show the color images of the three films and their blue shifts dependent on the VIS wavelength at the same irradiation time of 10 min and the same irradiance of 1.5 W/cm^2^. Besides the general regulation that all blue shifts of three films increase corresponding with the increasing incident wavelength, the most important and interesting demonstration is the remarkable bigger blue shift value at the plasmonic excitation wavelength of 529 nm for the AuNPs film and 423 nm for the AgNPs than that at the neighbor wavelengths. Figure 5b shows the light-to-thermal responsive characters of all three films much more clearly, the trends of which is the same as that of Fig. 5a. It is notable that the noble nanoparticles can absorb and constrain light around them to cause the film temperature sharply rising particularly at the plasmonic excitation wavelength. For a better exhibition of the dynamical VIS-stimuli chromic responsive performance of the AuNPs film at the plasmonic excitation wavelength, the Supplementary Fig. S5a,b show its color images and temperature, and then Fig. 5c,d show its blue shifts and length shrinkage percentages dependent on irradiation time. All above mentioned figures display that the film with AuNPs under the plasmonic exciting VIS (528 nm) has the much stronger color changes, temperature increments, blue shifts and length shrinkage percentages at the same time than the common VIS (420 nm). It fully infers that the plasmonic effect can improve and tune the photoresponsivity of the AuNPs film. As similar with the thermal and NIR stimuli, the plasmonic excitation VIS can also induce the state transition of the AuNPs film below and above LCST which the common VIS cannot due to its weak ligth-to-thermal conversion. The reason is still that AuNPs can promote the light-to-thermal conversion only under the irradiation of the plasmonic excitation VIS. The Supplementary Fig. S6 shows the same results for the AgNPs film and proves again that the plasmonic excitation VIS (423 nm) of AgNPs can also tune the conformation state (LSCT) and improve the photoresponsive sensitivity of the PNcM film.

### Repeatability experiments

The repeat experiments on thermosensitive were conducted that the films were soaked in DI water. The water was heated to 42 °C to record the reflection spectra and then cooled to 28 °C to record the spectra. The recycling experiments conducted five times. The repeat experiments on photosensitive were conducted that the films were illuminated under 973 nm light for 30 min to record the reflection spectra and shaded for 10 min to record the spectra. The recycling experiments conducted for five times to confirm the repeatability of the films.

**Figure 6:**
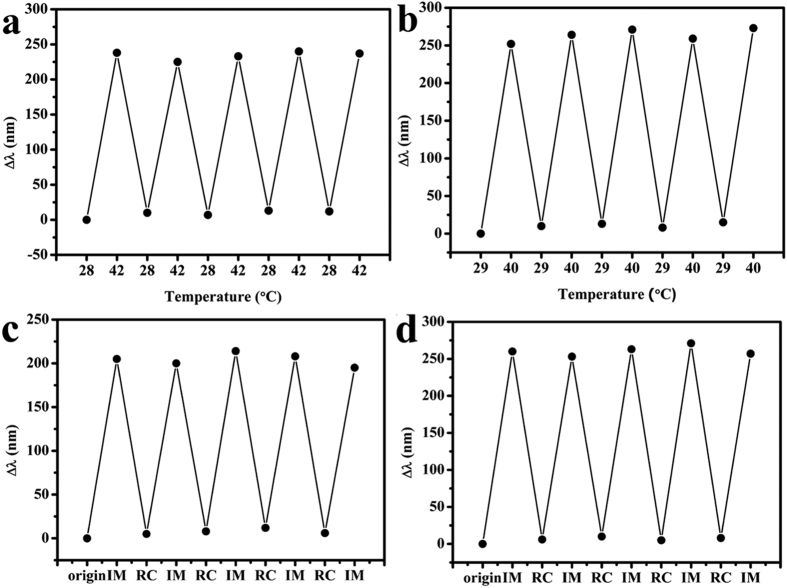
Repeatability experiments on thermochromics of (**a**) PNcM and (**b**) AuNPs@PNcM films recycling the temperature of 28 and 42 °C (AuNPs@PNcM is 40 °C) for 5 times; repeatability experiments on photochromic of (**c**) PNcM and (**d**) AuNPs@PNcM films recycling the illumination of 973 nm NIR for 30 min and recover for 5 times; IM is short for illuminating and RC is short for recovering.

At last, the results of the repeatable tests are shown in Fig. 6, which certainly shows that both films are repeatable and reversible regardless of the temperature and NIR-stimuli chromic responses. As for the VIS-response, the AuNPs film is also repeatability.

## Materials and Methods

### Chemical and Equipment

Tetraethoxysilane (TEOS), ammonium hydroxide (28%), NIPAm, Ethanol, N,N,N′,N′-Tetramethylethylenediamine (TEMED), methacrylic acid (AAc), Bis-acrylamide (BIS), ammonium persulfate (APS) and methacrylic acid (MAAc) were obtained from Sigma. Chloroauric acid, Argentum nitrate, Sodium borohydride and Sodium citrate were purchased from Sinopharm Chemical Reagent Co., Ltd. (Shanghai China). All these chemicals were analytical grade and used as received without further purification. DI water was used throughout the experiments. The SEM images were obtained with a FEISirion200 SEM. Before imaging, the samples were arc-coated with a thin gold film. The optical response of the photonic films was measured by using an OceanOptics Maya 2000 fiber optic spectrometer. The color images were taken by Meizu Mx4 camera. The monochromatic lights were obtained from the PLS-SXE300 Xenon Light Source with the optical filters (wavelengths are 365, 420, 528, 670, 748, 847 and 973 nm). The adsorption spectra were recorded on a Quawell Ultraviolet spectrophotometer and Lambda 750 PerkinElmer spectrophotometer. And the irradiation of all the incident lights was recorded by the PG2000-pro optical spectral measurement.

### Synthesis of Silica colloidal crystal

Briefly, 6.25 ml ammonia hydroxide and 60 ml anhydrous ethanol were mixed in a 100 ml flask, stirred intensively with a magnetic beater at 298 K keeping the speed of 1000 rmp. And then 2 ml tetraethoxylsilane (TEOS) was added. What the most important point is that the mixed solution should be stirred at the speed of 1000 rmp for 120 seconds first and then at the speed of 400 rmp for 24 hours. The colloidal particles were obtained by centrifugation and ultrasonic cleaning 4 times with anhydrous ethanol to remove impurities in the colloidal particles. The pretreatment SiO_2_ particles were diluted by anhydrous ethanol to a weight concentration of 0.7% and then transferred into clean 10 ml vials with a 1 cm × 4 cm glass slide. About 7 days later, when ethanol evaporated, silica colloidal crystal templates were formed on the both sides of the slide.

### Synthesize of AuNPs

1.0 wt% of the HAuCl_4_ solution A and 0.1 wt% of the sodium citrate solutions B were prepared. 200 μl of solution A was added into 50 ml DI water and then was heated to be boiling with the stirring rate of 600 rmp. 2 ml of the solution B was rapidly injected into the mixing solution keeping the temperature and stirring rate. After 10 min, the solution turned into wine red and the reaction was finished. The AuNPs solution was centrifugal cleaning for 30 min with DI water with the speed of 12000 rmp for 4 times.

### Synthesize of AgNPs

A 20 mL of 1.0 wt% citrate solution and a 75 mL of water were added in a round bottom flask and the mixture was heated to 70 °C for 15 min. After that, 1.7 mL of 1.0 wt% AgNO_3_ solution was introduced to the mixture, followed by the quick addition of 2 mL of 0.1 wt% freshly prepared NaBH_4_ solution. The reaction solution was kept at 70 °C under vigorous stirring for 1 h and cooled to room temperature. Water was added to bring the volume of the dispersion to 100 ml. The resulting AgNPs were used as starter seeds. The diameter of the AuNPs is about 13 ± 1.5 nm. Next, 10.0 mL of starter seeds solution was added while vigorously mechanical stirring, followed by the addition of 1.7 mL of 1.0% AgNO_3_ solution. Stirring continued for 1 h while keeping reflux and cooled to room temperature. AgNPs synthesized in this way were spherical with an average size of 20 nm. The AgNPs solution was centrifugal cleaning for 20 min with DI water with the speed of 8000 rmp for 4 times. The diameter of the AgNPs is about 20 ± 2.5 nm

### Synthesis of PNcM with AuNPs/AgNPs thin film

The monomer concentrations of NIPA and MAAc were 1.5 M and 0.2 M, respectively, and 10 mol% of BIS, 1 mol% of an initiator, TEMED, versus the total monomer concentration (1.7 M) and 0.05 mM 13 nm gold nanoparticles solution/20 nm silver nanoparticles solution were dissolved in DI water. The concentration of the AuNPs dispersing in the PNcM film is 3.65 × 10^9^ nanoparticles/cm^3^ and that of AgNPs is 2.4 × 10^9^ nanoparticles/cm^3^. 5 wt% of APS solution was prepared. 200 ul of the monomer solution and 5 ul of the APS solution were mixed in a 1.5 ml tube and 10 ul of the mixing solution was injected rapidly into the gap between the glass sheet with PC structure and PMMA sheet forming the Sandwich fabric. After 30 min at room temperature, the Sandwich fabric was immersed in 1.2% hydrofluoric acid for at least 12 h to obtain the inverse opal free-standing thin film. And the film was washed with DI water for 6 times and placed in DI water for testing.

### Thermoresponse measurements

First, the films were respectively soaked in insulation culture dishes which were filled with water at a certain temperature (varied from 28 to 42 °C). Then, the reflective spectra of the films at the midpoint were measured by the OceanOptics Maya 2000 fiber optic spectrometer. At last, the color photos and length of the films were recorded by a camera with a length reference object. For the experiment on temperature gradient, the PNcM film was tested only. The film was soaked in 28 °C water and then different volume of 50 °C water was added to one side of the film rapidly. We used the camera to take photos and the infrared thermometer to record the initial temperature. The transient temperature of the other side of the film was calculated.

### Photoresponse measurements

For the NIR-chromic behaviors, we soaked the film in a small amount of water in the culture dish and used xenon light with 748, 847 and 973 nm optical filters respectively to illuminate the film with a 3 mm focus in diameter. The illumination distance was about 10 cm and the irradiance of all incidents light was 1.5 W/cm^2^ which was measured and controlled by the PG2000-pro optical spectrum measurement. After irradiation 10 min, we turned off the xenon light and recorded the reflective spectrum immediately. At the same time, pictures, length and temperature were acquired as the way of thermochromics test. For the test on 973 nm only, the films were illuminated for 0, 5, 10, 15, 20, 25 and 30 min. The experiments on VIS-chromic response are the same as those of NIR-chromic above. What is different is that we utilized the 365, 420, 528 and 670 nm optical filters to get the visible monochromatic incident light. And the experiments on plasmonic effect with 528 and 420 nm incident light were illuminated for 0 to 90 min with 5 min interval.

## Conclusion

In conclusion, a sensitive, reversible and repeatable thermo- and photo-stimuli chromic responsive PNcM film with the inverse opal structure and the noble nanoparticles addition is developed for biomimetic chameleon. The methodology provides an easy approach for vivid, rapid and autonomic colorimetric response to the thermo- and photo-stimuli like a chameleon. The attractive creativity is to take use of the plasmonic nanoparticles like AuNPs and AgNPs to improve the sensitivity of the responsive behaviors and control the LCST of PNcM polymer by tuning the polymer chain conformation transition for all three conditions of temperature variation, NIR and VIS irradiation. Furthermore, it is fascinating that the biomimetic film can present all colors over the entire VIS spectrum by the addition of different type, size and morphology noble nanoparticles to tune the plasmonic excitation wavelength in the entire VIS spectrum. In addition, if raising the incident intensity to increasing the photo-responsive efficiency, this biomimetic film may provide an inspiration for energy storage. Therefore, this biomimetic film will be of great significance for new biosensors, communication mode, energy storage, camouflage and stealth in the future.

## Additional Information

**How to cite this article**: Yan, Y. *et al.* Plasmonic nanoparticles tuned thermal sensitive photonic polymer for biomimetic chameleon. *Sci. Rep.* **6**, 31328; doi: 10.1038/srep31328 (2016).

## Supplementary Material

Supplementary Information

## Figures and Tables

**Figure 1:**
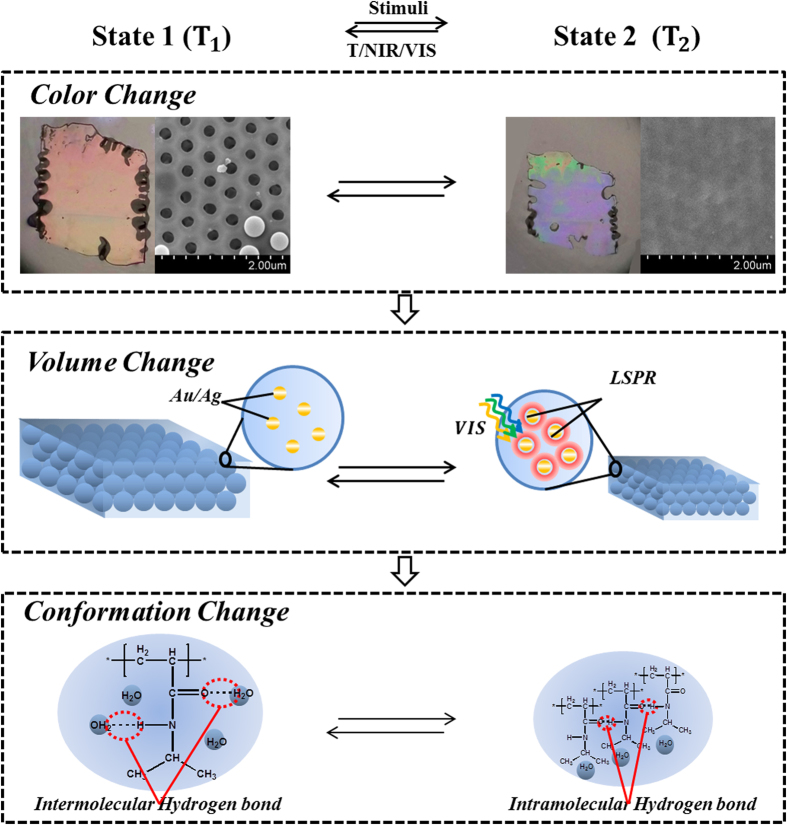
Scheme of the biomimetic chameleon responding to surroundings temperature, near infrared and visible light with color, volume and conformation variation tuned by plasmonic nanoparticles.
