# Supplementary material for: Plasmonic nanoparticles tuned thermal sensitive photonic polymer for biomimetic chameleon
Source: Sci Rep. 2016 Aug 9;6:31328. doi: 10.1038/srep31328 (PMC4977561; doi:10.1038/srep31328)
Supplement: Supplementary Information [file srep31328-s1.pdf]

Supporting Information

Plasmonic nanoparticles tuned thermal sensitive photonic polymer for biomimetic chameleon

Yang Yan[1], Lin Liu[1], Zihe Cai[1], Jiwen Xu[2], Zhou Xu[1], Di Zhang[1] and Xiaobin Hu*[1]

[1]State Key Laboratory of Metal Matrix Composites, Shanghai JiaoTong University, Shanghai 200240, People's Republic of China

[2]Guangxi Key Laboratory of Information Materials, Guilin University of Electronic Technology, Guilin 541004, People's Republic of China

Corresponding author: Xiaobin Hu

E-mail: hxb@sjtu.edu.cn

Figures Section

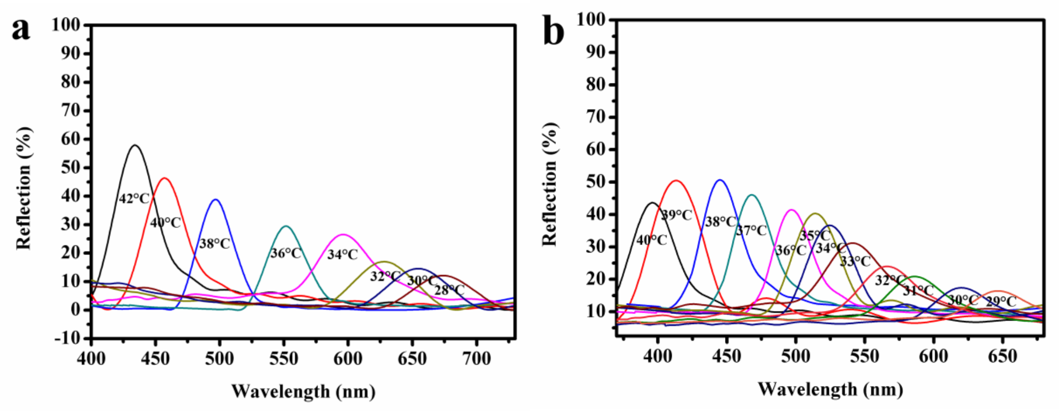

Figure S1. Reflective spectra of a) PNcM film and b) AuNPs@PNcM film dependent on temperature.

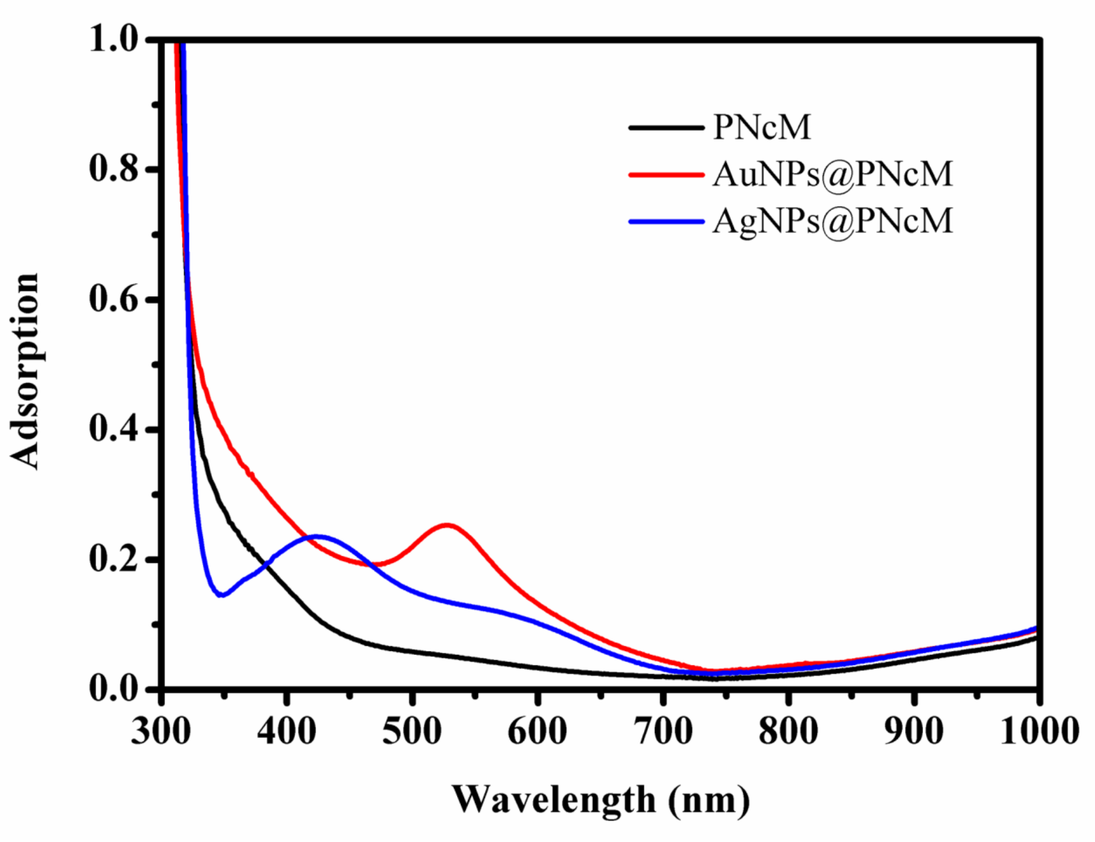

Figure S2. The adsorption spectrums of PNcM, AuNPs@PNcM and AgNPs@PNcM films without photonic crystal structure.

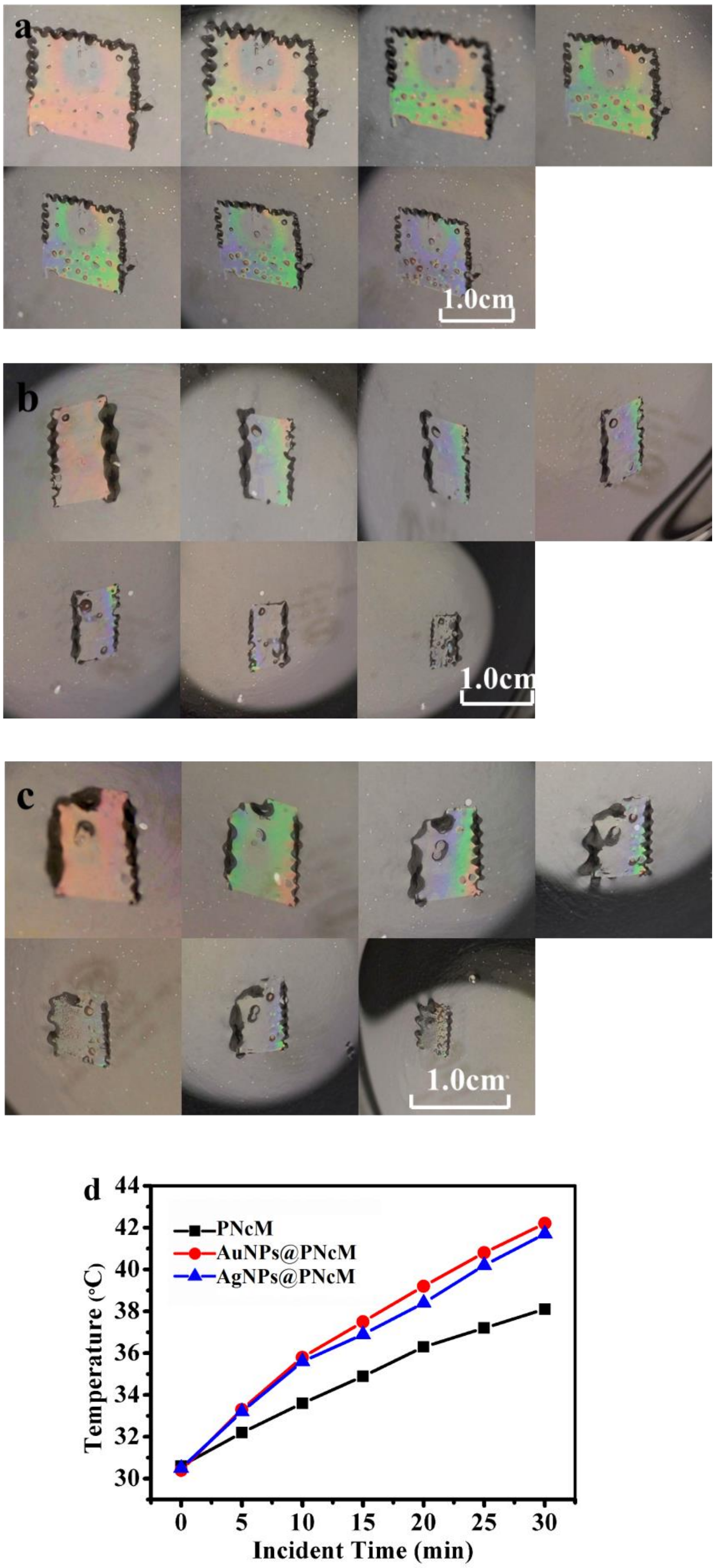

Figure S3. Color change of a) PNcM, b) AuNPs@PNcM and c) AgNPs@PNcM films and d) temperature of the three films illuminated by 973 nm NIR dependent on the incident time from 0, 5, 10, 15, 20, 25, 30 min, respectively

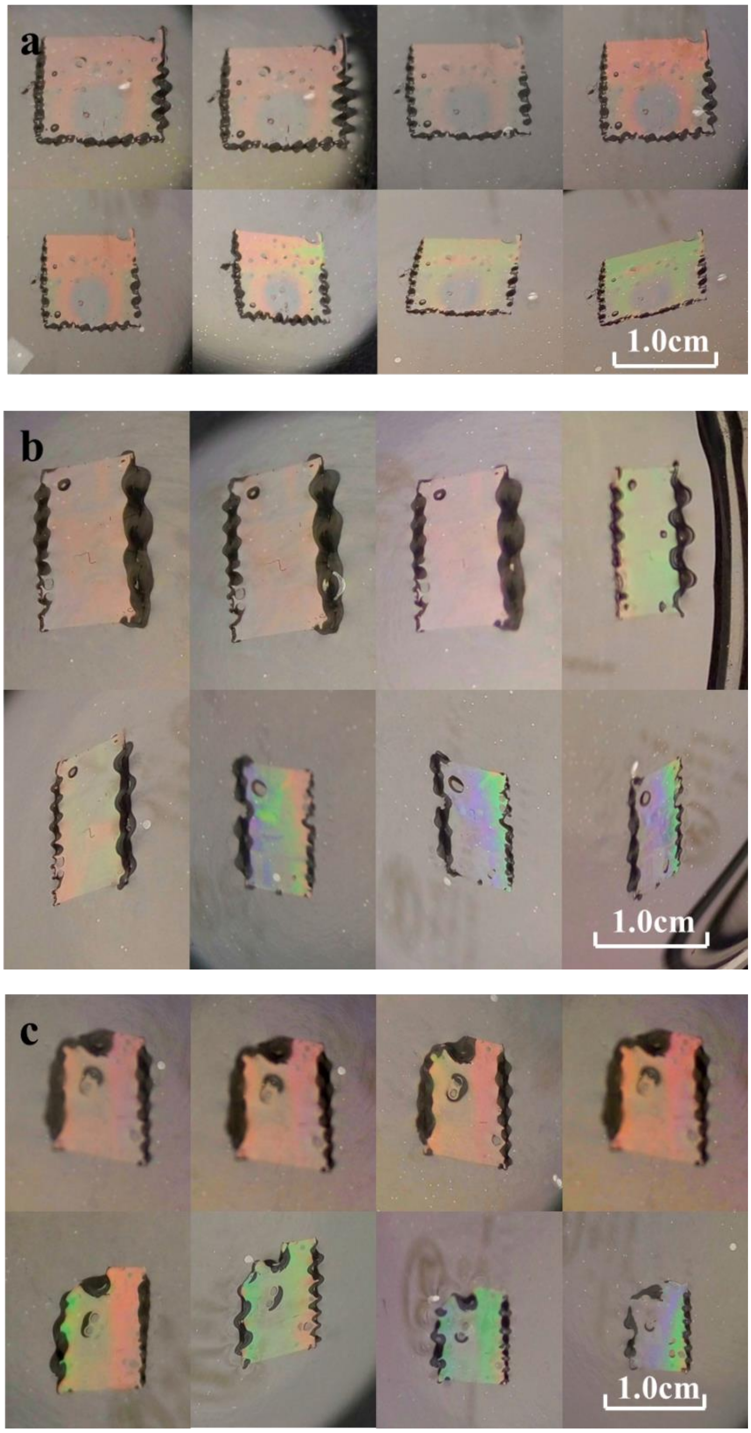

Figure S4. Color change of a) PNcM, b) AuNPs@PNcM and c) AgNPs@PNcM films dependent on the incident wavelength of blank, 365, 420, 528, 670, 748, 847 and 973 nm illumination for 10min.

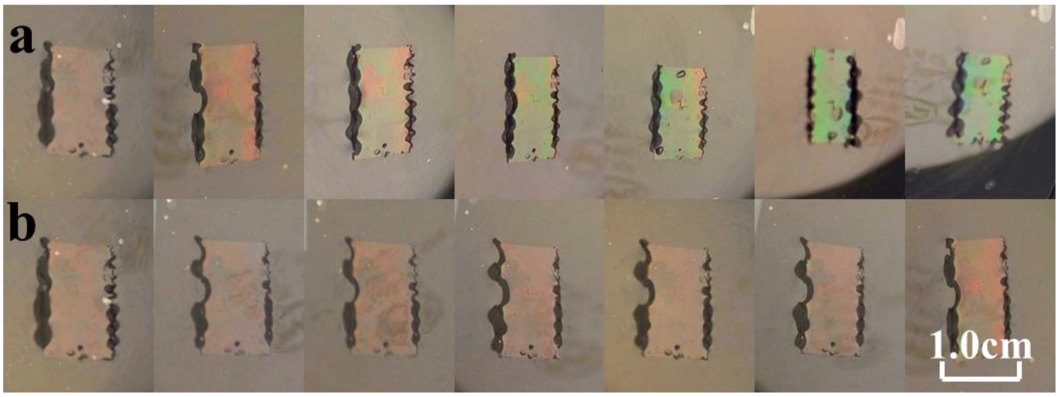

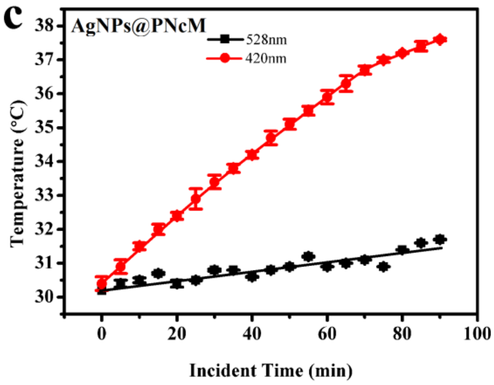

Figure S5. Color change of the AuNPs@PNcM film under a) 528 nm and b) 420 nm incident VIS light illumination for 0, 5, 10, 15, 20, 25 and 30 min, and c) temperature variation of the AuNPs@PNcM film under 528 nm and 420 nm dependent on the illumination time from 0 to 90 min with 5 min interval, respectively

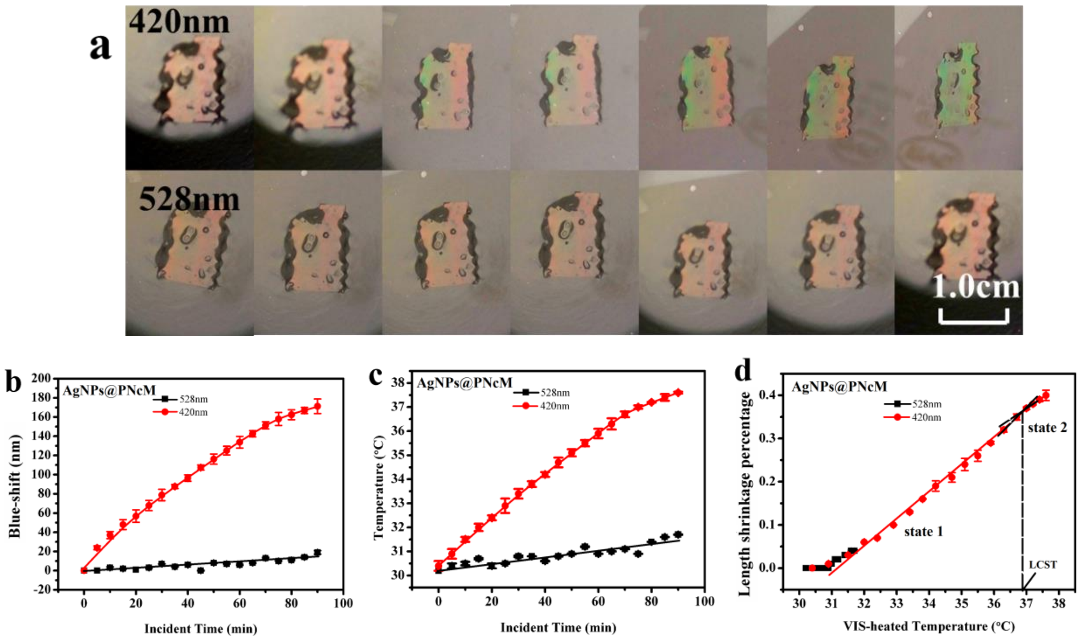

Figure S6. Vis-stimuli chromic responses of AgNPs@PNcM film of a) color variation (illumination for 0, 5, 10, 15, 20, 25 and 30 min), b) blue-shift, c) temperature increment and d) length shrinkage percentage under 528 and 420 nm dependent on the incident time from 0 to 90 min with 5 min interval.
